# Modeling the Role of pH on Baltic Sea Cyanobacteria

**DOI:** 10.3390/life5021204

**Published:** 2015-03-30

**Authors:** Jana Hinners, Richard Hofmeister, Inga Hense

**Affiliations:** 1 Institute for Hydrobiology and Fisheries Science, Center for Earth System Research and Sustainability, University of Hamburg, Große Elbstraße 133, 22767 Hamburg, Germany; E-Mails: Richard.Hofmeister@uni-hamburg.de (R.H.); Inga.Hense@uni-hamburg.de (I.H.); 2 Helmholtz-Zentrum Geesthacht, Max-Planck-Straße 1, 21502 Geesthacht, Germany

**Keywords:** Baltic Sea, cyanobacteria, climate change, phytoplankton, pH, ocean acidification

## Abstract

We simulate pH-dependent growth of cyanobacteria with an ecosystem model for the central Baltic Sea. Four model components—a life cycle model of cyanobacteria, a biogeochemical model, a carbonate chemistry model and a water column model—are coupled via the framework for aquatic biogeochemical models. The coupled model is forced by the output of a regional climate model, based on the A1B emission scenario. With this coupled model, we perform simulations for the period 1968–2098. Our simulation experiments suggest that in the future, cyanobacteria growth is hardly affected by the projected pH decrease. However, in the simulation phase prior to 1980, cyanobacteria growth and N${}_{2}$-fixation are limited by the relatively high pH. The observed absence of cyanobacteria before the 1960s may thus be explained not only by lower eutrophication levels, but also by a higher alkalinity.

## 1. Introduction

During the past few decades, an increase of cyanobacteria blooms was detected in the Baltic Sea and generally attributed to a rise in sea surface temperature (SST) and eutrophication [1,2]. Both aspects were already addressed in modeling studies (e.g., [3,4,5]), but what is missing in projection studies are possible effects through pH changes. As experimental studies, e.g., [6,7,8,9], show that a decreasing pH affects cyanobacteria growth, as well, we will incorporate their findings into this model study for the central Baltic Sea.

Cyanobacteria accumulations can have severe effects on the ecosystem. They can clog the feeding apparatus of grazers, can have deleterious effects on higher trophic levels, also through cyanobacterial toxin production [10], and they can lead to oxygen deficiency while blooms are decomposed [11]. Apart from these biogeochemical effects, cyanobacteria also influence ocean physics [12]. Surface mats of cyanobacteria can increase the light absorption and surface albedo [13] and can dampen the influence of wind drag [14].

Changing climatic conditions in the future will likely cause changes in the cyanobacteria biomass. The temperature optimum for the growth of most cyanobacteria lies at ∼25 ${}^{\circ }\text{C}$ [15,16] or even higher [17], and thus, they can benefit from a warming climate. Projection scenarios suggest that cyanobacteria concentrations might be more than two-fold higher before the end of the 21st century due to higher SST; in addition, the start of the bloom might occur earlier in the year [5]. Regarding eutrophication, biogeochemical models project a moderate or a strong increase in cyanobacteria in the future, depending on the assumptions made concerning changes in nutrient discharges into the Baltic Sea [4]. However, none of the modeling studies account for the effects of ocean acidification.

Under business-as-usual conditions, the decrease in ocean pH is expected to sum up to 0.3 units until the year 2100 [18]. Projections by Omstedt *et al*. [19] show that acidification in the central Baltic Sea will continue with increasing atmospheric CO${}_{2}$ concentrations, and even eutrophication with enhanced primary production will not impede this process. The seasonal cycle might be amplified by increasing photosynthesis and respiration activities, however. At present, the seasonal fluctuations in the Baltic Sea are about 0.5 pH units with minimum values around 8.0 and maximum values around 8.5 [20]. In deeper layers, the pH fluctuations decline, and the pH is generally lower [21].

The effect of a decreasing pH has been experimentally investigated for different cyanobacteria taxa. Laboratory experiments with the (sub-)tropical, non-heterocystous *Trichodesmium* are in agreement with an increasing growth for a higher CO${}_{2}$ concentration and a lower pH [22,23,24]. However, for one of the common cyanobacteria species in the Baltic Sea, *Nodularia spumigena*, a reduced cell division rate and nitrogen fixation rate were reported under acidified conditions [7,9]. Contradictory findings for the same species [8] might be due to a different experimental set up [9]. Why *N. spumigena* might not benefit from more acidified conditions could be explained by its natural growth conditions [7]. High primary production in the low buffered Baltic Sea environment can cause seasonally and locally alkaline conditions to which the cyanobacteria could be adapted; e.g., Ploug [25] reported pH values up to nine in cyanobacterial microenvironments. Moreover, Eichner *et al*. [9] proposed that the magnitude of the CO${}_{2}$ response might be correlated to the energy limitation and that heterocystous *N. spumigena* might have lower energy demands than *Trichodesmium*, since no daily synthesis and degradation of nitrogenase and storage products are needed. Apart from *N. spumigena*, another common Baltic Sea cyanobacterium, *Aphanizomenon flos-aquae*, showed a decreasing growth for a pH lower than pH 8, as well [6]. Based on the insights gained from the laboratory experiments [6,7,9], we may expect a potential decrease of cyanobacteria concentrations in the Baltic Sea in the future due to more acidified conditions.

In this study, we incorporate the pH-dependence of cyanobacteria growth in an ecosystem model for the central Baltic Sea. The pH-dependence of cyanobacteria, obtained from experimental data, is integrated into a cyanobacteria life cycle model, which is coupled to a biogeochemical model, to a water column model and to a carbonate chemistry model. Our model system is two-way coupled; we allow the ecosystem to alter the carbonate chemistry and consider the feedback between the carbonate chemistry and cyanobacteria. In this way, our model does not only include a decreasing pH, but also seasonal pH alterations due to biogeochemical processes. We perform simulations for the period 1968–2098 in order to answer the following question: How do long-term trends and the seasonal variability of pH influence cyanobacteria growth?

## 2. Model Design

### 2.1. The Coupled Physical-Biogeochemical Model

The physical model GOTM (general ocean turbulence model; [26]), the biogeochemical model (ERGOM; [27]), the cyanobacteria life cycle model (CLC; [28]) as well as a carbonate system model (CM; [29]) are coupled via the Framework for Aquatic Biogeochemical Models (FABM) [30]. All model components are explained in more detail.

#### 2.1.1. Physical Model and Atmospheric Forcing

The physical basis for our model system is the one-dimensional water column model, GOTM. It describes the most important hydrodynamic and thermodynamic processes in the water column by vertical fluxes of momentum, heat and salt [26]. As in the study by Hense and Burchard [31], we use a time step of 900 s for the simulation and a water depth of 250 m, which is divided into 100 layers in this study. The layer thickness varies depending on the water depth between 0.7 m at the surface, 3.7 m in medium depth layers and 1.6 m at the bottom.

Our model simulations begin in 1958 and continue through 2098. The first decade of our simulations will be regarded as a spin-up phase and is excluded from analysis. In order to simulate the conditions in the central Baltic Sea (57.3 ${}^{\circ }\text{N}$, 20 ${}^{\circ }\text{E}$) in our model, we use six-hourly atmospheric forcing (2-m air and dew point temperature, 10-m zonal and meridional wind velocities, cloud cover and precipitation). The forcing fields stem from a downscaling climate model run that has been carried out with the regional Baltic Sea coupled atmosphere ocean model RCAO [4,32]; this regional model makes use of the atmospheric forcing fields of the global HadCM3-A1B-scenario [33].

#### 2.1.2. Biogeochemical Model

The biogeochemical environment of our model is provided by the ecosystem model, ERGOM [27], containing cyanobacteria, diatoms, flagellates, zooplankton, nitrate, ammonium, dissolved inorganic phosphorus (DIP), detritus and oxygen. The primary producers are represented by diatoms (large phytoplankton, growing fast under nutrient rich conditions), flagellates (small phytoplankton, advantaged under lower nutrient concentration) and cyanobacteria [34]. Yet, similar to Hense and Burchard [31] and Hense *et al*. [5], we replaced the cyanobacteria compartment by a cyanobacteria life cycle model [28].

The growth rate of diatoms and flagellates depends on DIN and DIP (dissolved inorganic nitrogen and phosphorus); cyanobacteria can take up DIN, but they are also able to fix dinitrogen gas [35,36]. However, N${}_{2}$-fixation requires much more energy and thus usually occurs under low DIN concentrations. We assume that cyanobacteria are not limited by phosphorus, as their phosphorous demand is accommodated via the use of organic phosphorous [37,38]; additionally, they show a high plasticity in cellular stoichiometry [39]. The growth of all primary producers depends on solar radiation and temperature; cyanobacteria growth is additionally pH dependent. Diatoms and flagellates can be consumed by zooplankton. Dead organic matter is remineralized in the water column or settles down, where it is either remineralized, resuspended or buried [34]. The oxygen and phosphorus development is coupled to the nitrogen cycling via constant stoichiometric ratios and controls further processes [34].

All biological variables are allowed to feed back on temperature through light absorption, which leads generally to a warming of the surface and cooling of the subsurface layer [40].

### 2.2. Cyanobacteria Life Cycle Model

#### 2.2.1. General Model Description

We use the same cyanobacteria life cycle model as in previous studies [5,28,31]: The cyanobacteria life cycle stages are described by their energy and nitrogen quota. Vegetative cells (VEG) take up dissolved inorganic nitrogen (DIN) and have a high energy and a high nitrogen quota. When the available DIN is exhausted and the nitrogen quota sinks, a biomass flux into the model compartment HET (vegetative cell with heterocysts) takes place. The model compartment HET is characterized by a high energy, but a low nitrogen quota; the nitrogen supply occurs through the high energy demanding process of nitrogen fixation. Decreasing solar radiation in autumn causes a decreasing energy quota, and HETs transform into akinetes (AKI) that have a low energy and nitrogen quota. The akinetes sink to the bottom, where they slowly take up DIN. As soon as the nitrogen quota is above a certain threshold, the akinetes turn into recruiting cells (REC) that have a low energy, but high nitrogen quota. RECs have an upward velocity, and at surface, they can fill the energy quota and, thus, become VEGs again. A striking feature of this model system is that it accounts for the feedbacks related to the life cycle processes. Higher growth of VEGs, and particularly of HETs, will lead to more AKIs, which, in turn, will trigger bloom formation again in the following year.

The mortality of the growing life cycle stages is described by a linear term [28]. VEGs and HETs are positively buoyant, whereas AKIs sink to the bottom of the water column and RECs have an upward velocity. All processes of the life cycle are directly or indirectly dependent on temperature and light [5]. We use the same model dependencies and parameters as in previous studies, except that we add a pH-dependence for the growth of VEGs and HETs (see the next section). Our maximal metabolic rate for VEGs and HETs is ${\text{ω}}_{0}=0.334\;{\text{day}}^{-1}$ and for AKIs and RECs ${\text{ω}}_{0}=0.242\;{\text{day}}^{-1}$.

The life cycle model of cyanobacteria has been applied in previous studies to the central Baltic Sea and shows a realistic seasonal cycle compared to observations [40].

#### 2.2.2. pH-Dependent Cyanobacteria Growth

Experimental studies by Eichner *et al*. [9] and Yamamoto and Nakahara [6] show a decreasing growth rate for cyanobacteria under more acidified conditions than today. While the former [9] use specifically a species from the Baltic Sea (*Nodularia spumigena*), the latter [6] utilize a cyanobacteria species (*Aphanizomenon flos-aquae*) that does not originally stem from the Baltic Sea, but is nevertheless dominant there. We are aware that there is some uncertainty related to this approach, because we cannot exclude that species of the same taxa can genetically adapt to the local conditions. However, the study by Yamamoto and Nakahara [6] is one of the few studies, in which the growth rate over a wide range of pH has been determined. In addition, the pH-range for the growth of all three common Baltic Sea species, *Nodularia spumigena*, *Aphanizomenon flos-aquae* and *Anabaena sp.*, seems to be not too different compared to these species that occur in other aquatic systems.

**Figure 1:**
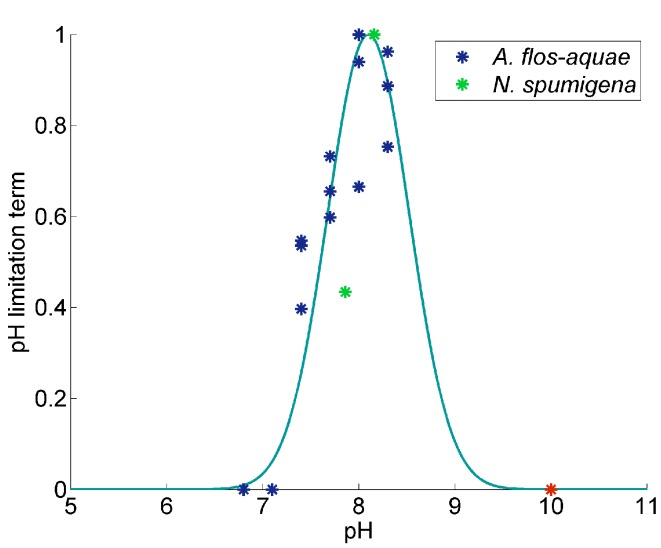
A functional fit through the data points that stem from the laboratory experiments with *A. flos-aquae* (blue) [6] and *N. spumigena* (green) [9]. To depict the data in one graph and to extract the limitation function for our model, we divided the species-specific growth rate by the respective maximum growth rate. Moreover, since no growth is expected above pH 10 (e.g., [41]), we added this data point (red).

Both laboratory experiments provide the basis to describe the pH-dependent growth of cyanobacteria in our model study. We extracted the data with the help of the Internet-based Webplotdigitizer (http://arohatgi.info/WebPlotDigitizer) and fit a Gauss curve through the data points. To determine the pH-limitation function for growth, we first calculated the “relative growth rate” of both species by dividing the species-specific growth rate by the respective maximum growth rate. There is common agreement that the cyanobacterial growth declines to zero at a pH > 9.5 [41,42] and thus, we included this feature, as well. The observations and our functional fit are depicted in Figure 1.

Our fit results in the following equation for the pH-limitation term, which we apply for the growth rate, as well as the nitrogen fixation rate for VEG and HET:

(1) $$\begin{array}{c} f_{\text{pH}}=\exp\left(\frac{-{\left(\text{pH}-{\text{pH}}_{\mathrm{opt}}\right)}^{2}}{{{\text{pH}}_{\mathrm{slope}}}^{2}}\right) \end{array}$$

with ${\text{pH}}_{\mathrm{opt}}=8.1$ and ${\text{pH}}_{\mathrm{slope}}=0.6$ (RMS=0.16).

### 2.3. Model of the Carbonate System

The carbonate system is computed using the model by Blackford and Gilbert [29]. The model calculates the composition of DIC (carbonate, bicarbonate) and resulting pH taking into account temperature, salinity, depth, carbon dioxide concentrations, as well as total alkalinity ($A_{T}$). Similar to Blackford and Gilbert [29], the latter ($A_{T}$) is parameterized from salinity fields. Specifically, $A_{T}$ and salinity *S* are assumed to be linearly correlated as $A_{T}=25.3\;S+1470\;\left[\mathrm{\mu mol}\;{\mathrm{kg}}^{-1}\right]$, following Beldowski *et al*. [43]. Thus, in this approach, alkalinity is not a prognostic variable, and several feedbacks related to alkalinity cannot be captured.

The carbonate chemistry model is forced by annual mean atmospheric carbon dioxide concentrations from the Special Report on Emissions Scenarios (SRES) A1B emission scenario [44].

DIC is taken up by autotrophs and produced via respiration or remineralization (in the CLC and ERGOM model). Thus, the ecosystem alters the carbonate chemistry, and *vice versa*, the carbonate chemistry affects the ecosystem, as the growth of cyanobacteria is pH dependent.

### 2.4. Model Experiments

We perform three different model experiments in order to investigate the influence of long-term trends and the seasonality of the pH on cyanobacteria. In our first experiment (PH-VAR), we consider the effects of pH on cyanobacteria growth; the carbonate system and, thus, the temporal variability of pH is resolved. In the second model run, the growth of cyanobacteria is not limited by pH (NOLIM); *i.e.*, $f_{\text{pH}}=1.0$. The third experiment excludes the diurnal and seasonal signal of pH (PH-YMEAN). In the latter, the annually-averaged results of pH from the model run (PH-VAR) are used as a forcing (instead of computing the carbonate system with the carbonate chemistry model (CM)).

## 3. Results and Discussion

The first ten years are used as spin-up, and we only analyze the model results of the cyanobacteria projection for the years between 1968 and 2098. Climate projections cannot reproduce a specific sequence of years or year-to-year variability in the past or future. Since biological systems have a memory and are thus sensitive to past variations in the environment [5], we do not focus on individual years or decadal variability, but on processes that are related to long-term trends or seasonal variability.

### 3.1. Seasonal Variability of Life Cycle Stages and pH

**Figure 2:**
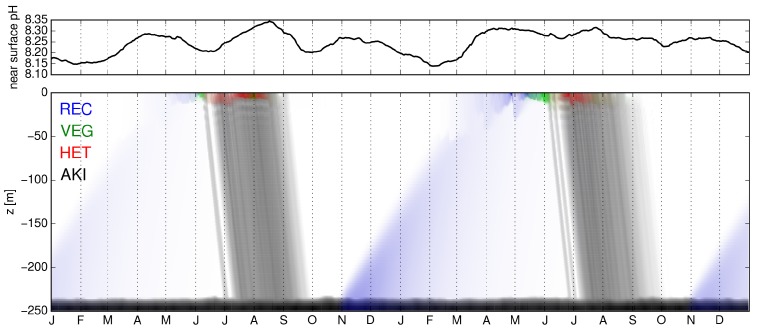
(**Top**) Surface pH within two years. (**Bottom**) Succession of cyanobacteria life cycle stages within the same time period. The concentration of each life cycle stage is visualized by the color intensity, *i.e.*, light denotes low, saturated color high concentrations. For the concentrations of akinetes, the color intensity scales logarithmically for concentrations above 10${}^{-5}$ mmol N m${}^{-3}$.

Similar to previous studies, a succession of the four different life cycle stages over the course of a year takes place (Figure 2, bottom). During spring, RECs ascend to the surface, where they transform into VEGs. These turn into HETs as soon as DIN is exhausted. HETs are able to fix dinitrogen gas and form a dense bloom at the surface in summer before they transform in fall into AKIs, which sink to the bottom. At the bottom of the sea, AKIs build a reservoir from which new RECs are formed in the following spring. The dominant life cycle stage at the surface is HET, and it is also this stage that has the largest effect on pH.

Over the course of the year the sea surface pH experiences strong changes (Figure 2, top). During the phytoplankton spring bloom (not shown), CO${}_{2}$ is taken up and the pH increases. In June and July, the decomposition of the spring bloom causes a pH decrease, before the growth, and thus, CO${}_{2}$ uptake of HET during summer leads to an increasing pH again. After the summer bloom, a drop in pH takes place when organic matter is remineralized. This drop is followed by a small increase of pH in autumn due to enhanced phytoplankton growth. During the winter, decomposition leads to a decrease of pH with the lowest value before the spring bloom the following year. These seasonal pH fluctuations occur within a range of ∼0.3 pH units, which is slightly lower than measured pH fluctuations in the central Baltic Sea [20]. This feature also appears in other modeling studies (e.g., [45]) and can be related, for example, to the use of fixed stoichiometric ratios in phytoplankton, incorrect alkalinity discharges from rivers or missing details in the model description of the carbonate chemistry. In addition, elevated primary production and remineralization due to high nutrient concentrations can also amplify the seasonal variations in pH (see [19]).

Differences in pH and concentrations of the life cycle stages between the years are due to the interannually varying atmospheric forcing.

### 3.2. Projection of Cyanobacteria Abundance

**Figure 3:**
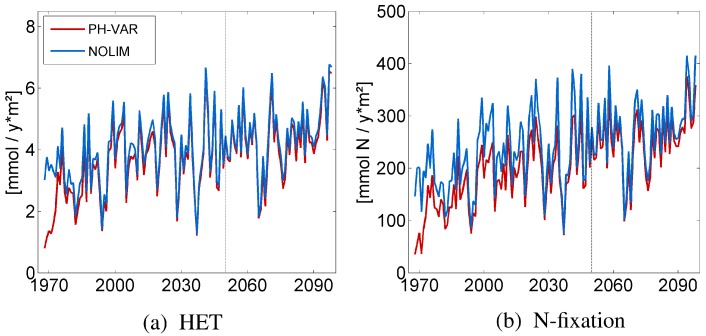
Time series between 1968–2098 of the vertically- integrated annual mean concentration of heterocyst (HET) (**a**) and the vertically integrated annual mean N${}_{2}$-fixation rate (**b**), for the PH-VAR and the NOLIM (not limited) model run. The optimal annual mean pH is reached around year 2050 (dashed line).

**Figure 4:**
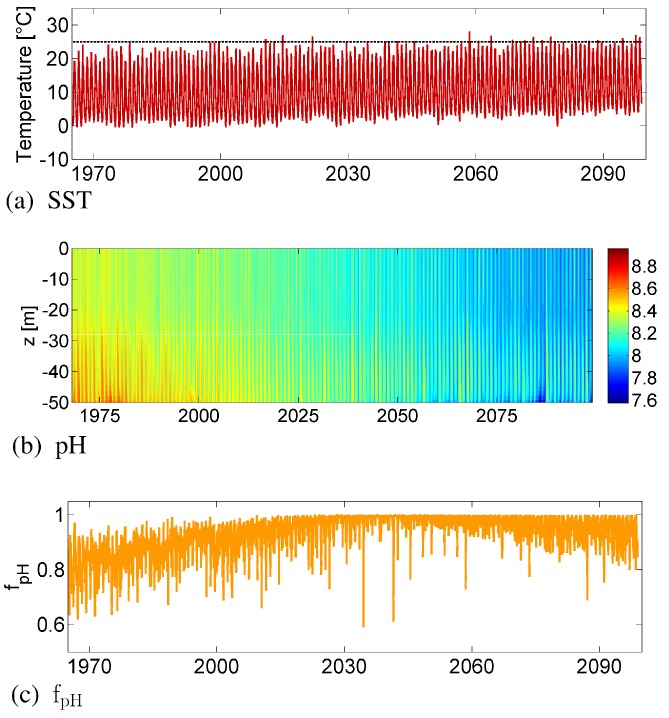
Time series of SST (**a**) and the pH in the water column (**b**). The dashed line in (a) indicates the optimal temperature for growth. The cyanobacteria growth is linked to the pH by the limitation term $f_{\text{pH}}$ (**c**).

The projection in the model run PH-VAR shows an increase in the cyanobacteria concentrations over the time period of 130 years (Figure 3a, red line). Light conditions hardly change in the future [5], and so, we concentrate on the effect of temperature and pH. In Figure 4, the pH and temperature development are visualized for the simulation period, as well as the corresponding limitation term for pH ($f_{\text{pH}}$) and the growth maximum for temperature. The surface pH down to a 50-m depth shows seasonal fluctuations and a long-term decrease (Figure 4b), while the sea surface temperature (SST) increases (Figure 4a).

During the first 10 years, a low SST and a too high pH limit the growth of cyanobacteria and lead to comparatively low concentrations (Figure 3a). From 1980 onwards, the growth conditions improve, and cyanobacteria concentrations increase, as the temperature rises and the pH reaches the optimum of cyanobacteria growth of 8.1, *i.e.*, $f_{\text{pH}}$ is increasing (Figure 4c). As the SST is only rarely higher than 25 ${}^{\circ }$C (Figure 4a), growth is not affected. The pH continues to decrease, but even in the second half of the 21st century, the surface pH remains close to the optimum for cyanobacteria growth. Overall, the positive trend in the cyanobacteria concentrations is a result of growth and life cycle-related feedback mechanisms, as described in the previous study by Hense *et al*. [5].

To evaluate the effect of pH, we conducted a model run (NOLIM) in which we neglect the limitation of growth by pH (Figure 3a, blue line). This model run is similar to previous studies in which the response of cyanobacteria to warming has been investigated [5]. Compared to the model experiment PH-VAR, the cyanobacteria concentrations are only slightly higher, except for the first 10 years. The relatively high pH during the 1970s clearly determines the growth conditions of cyanobacteria. Lower cyanobacteria concentrations in the past were actually found in the Baltic Sea [2], specifically at the beginning of the 20 th  century, but they were not attributed to a higher pH. Instead, the increasing cyanobacteria abundance detected since the 1960s was interpreted as the consequence of eutrophication [2,39]. However, growth stimulation due to a decreasing pH could have contributed to rising cyanobacteria concentrations in the past, as well. The differences between NOLIM and PH-VAR vanish in the middle of the 21st century, where the pH is close to the optimum and SST is relatively high to support growth in summer. Only at the end of the simulation period, the differences between both model runs slightly increase because pH has passed the optimum and growth conditions are then sub-optimal. Comparing the nitrogen fixation rates in NOLIM and PH-VAR, the differences are more pronounced, but the trend is the same (Figure 3b).

Finally, we also looked at whether the seasonal cycle of HET concentrations is affected by changes in pH. Similar to previous studies [3,5], we see a shift towards an earlier blooming that is stimulated by higher temperature; the pH changes have no effect.

### 3.3. The Role of Seasonal pH Variability

**Figure 5:**
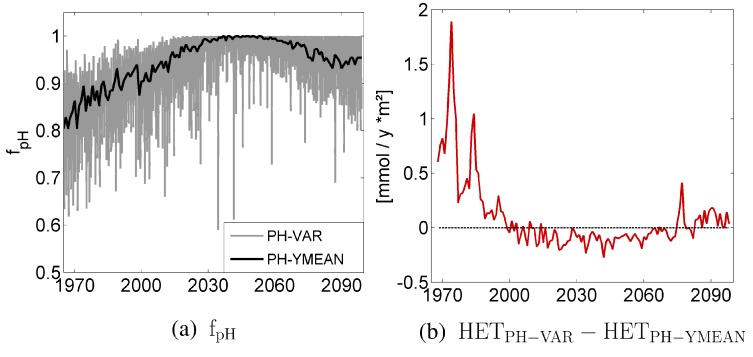
(**a**) The limitation term $f_{\text{pH}}$ for cyanobacteria growth at the surface in PH-VAR and PH-YMEAN. (**b**) The difference in annual mean vertically-integrated HET between PH-VAR and PH-YMEAN.

In order to investigate the effect of seasonality in pH on the cyanobacterial growth, we compare PH-VAR with the model run PH-YMEAN, where we use annually-averaged pH-values to force the growth of cyanobacteria. Figure 5a shows the pH limitation term $f_{\text{pH}}$ for both model experiments. As the pH decreases towards the optimum of cyanobacteria growth at pH 8.1 around year 2050, the limitation term approaches one in both model runs. Thus, the pH limitation of cyanobacteria growth decreases. After exceeding the optimum, the limitation increases again, visible in the lower $f_{\text{pH}}$.

Figure 5b shows the differences in HET biomass between PH-VAR and PH-YMEAN. Between 2000 and 2070, the difference between the concentrations of HET in both model runs is small; *i.e.*, obviously the seasonality has a minor effect on growth and bloom development of cyanobacteria under almost optimal pH conditions. However, any fluctuation in pH causes slightly more unfavorable conditions for cyanobacteria growth in PH-VAR compared to PH-YMEAN; this explains the marginally lower HET biomass in PH-VAR compared to PH-MEAN during this time period. In contrast, in the 1970s and close to the end of the simulation period, the seasonality of the pH allows for a higher HET biomass in PH-VAR compared to PH-YMEAN. This is due to the seasonal fluctuations of the pH, which occasionally causes a pH shift towards the optimum and, thus, stimulates cyanobacteria growth. During the first 10 years, however, a negative feedback mechanism prevails. If the environment is too alkaline, cyanobacterial CO${}_{2}$ uptake leads to a pH rise above the optimum pH value for growth and, thus, suboptimal growth conditions. In contrast, a positive feedback mechanism occurs for pH values that are lower than the optimum: cyanobacteria take up CO${}_{2}$ during growth, leading to an increase of pH and, thus, to better growth conditions. This positive feedback mechanism also explains why the differences in the model run PH-VAR and NOLIM in the second half of the 21st century are small.

### 3.4. Uncertainties and Model Limitations

All three model projections suggest an increase in the abundance of cyanobacteria due to a temperature rise and show that the influence of acidification on cyanobacteria is rather small. However, a few additional aspects need to be taken into account. First, even if the climate models correctly simulated the rise in temperature, the succession of warm and cold years is unknown. Due to the nonlinearity in biological systems, the response of cyanobacteria might be different than what the simulations show. Second, there are uncertainties with respect to the species-specific pH-dependence of the growth of cyanobacteria, as well as of other phytoplankton species that directly or indirectly interact. Additionally, the data we used to describe pH-dependent growth are based on laboratory experiments and can possibly contain laboratory artifacts; so far, mesocosm studies in a natural environment are missing to validate the experimental results. Third, it is unclear how fast species are able to genetically adapt. As shown by Lohbeck *et al*. [46], genetic adaptation to pH changes can occur on relatively short time scales (months to years). Fourth, there are a number of factors that can directly or indirectly affect the pH, but it is unknown how they evolve in the future. One of the most important ones is total alkalinity. Riverine and non-riverine alkalinity loads play an important role in the Baltic Sea [47] and may change in the future, but it is unclear in which direction. In addition, alkalinity is tightly coupled to salinity, which is, in turn, influenced by several processes on different temporal and spatial scales, like inflow events. Finally, it is unclear how the response of cyanobacteria will be in other regions of the Baltic Sea. The Baltic Sea is characterized by strong environmental gradients, and future changes will not be uniform. Indeed, previous projection scenarios for the Baltic Sea reveal significant differences in future pH changes between the basins [19]; changes in cyanobacteria concentrations and nitrogen fixation will thus likely vary among the different basins.

## 4. Summary and Conclusions

We integrated the pH dependence of cyanobacteria growth into an ecosystem model for the central Baltic Sea. Assuming a pH decrease following the A1B emission scenario, we performed simulations for the period 1968–2098. Our results suggest that in the future, the projected pH decrease has only a little impact on the intensity and timing of cyanobacteria blooms. Yet, the influence of the pH is stronger on N${}_{2}$-fixation than on cyanobacteria concentrations. Future laboratory and model studies should further address this issue to evaluate basin-wide effects on the nitrogen cycle.

The strongest effect of pH on cyanobacteria growth is visible in the simulation phase before 1980. During this phase, the growth of cyanobacteria is significantly limited by the relatively high pH compared to our reference model run without pH dependence. We therefore conclude that the observed absence of cyanobacteria blooms before the 1960s might not only be related to pre-eutrophied conditions, as has been previously speculated [2,39], but additionally to high pH-conditions. Hindcasts of Baltic Sea cyanobacteria for extended periods should therefore resolve the carbonate system and pH-dependent growth of cyanobacteria.
